# Diffusional kurtosis imaging of the corpus callosum in autism

**DOI:** 10.1186/s13229-018-0245-1

**Published:** 2018-12-13

**Authors:** Yu Veronica Sui, Jeffrey Donaldson, Laura Miles, James S. Babb, Francisco Xavier Castellanos, Mariana Lazar

**Affiliations:** 1 Department of Radiology, New York University School of Medicine, New York, NY USA; 2 Department of Child and Adolescent Psychiatry, Hassenfeld Children’s Hospital at NYU Langone, New York, NY USA; 3 Nathan Kline Institute for Psychiatric Research, Orangeburg, NY USA; 4 Center for Biomedical Imaging, NYU Langone Health, 660 First Ave, 4th floor, New York, NY 10016 USA

**Keywords:** Autism, Corpus callosum, Diffusional kurtosis imaging, Processing speed, Interhemispheric connectivity

## Abstract

**Background:**

The corpus callosum is implicated in the pathophysiology of autism spectrum disorder (ASD). However, specific structural deficits and underlying mechanisms are yet to be well defined.

**Methods:**

We employed diffusional kurtosis imaging (DKI) metrics to characterize white matter properties within five discrete segments of the corpus callosum in 17 typically developing (TD) adults and 16 age-matched participants with ASD without co-occurring intellectual disability (ID). The DKI metrics included axonal water fraction (*f*_axon_) and intra-axonal diffusivity (*D*_axon_), which reflect axonal density and caliber, and extra-axonal radial (RD_extra_) and axial (AD_extra_) diffusivities, which reflect myelination and microstructural organization of the extracellular space. The relationships between DKI metrics and processing speed, a cognitive feature known to be impaired in ASD, were also examined.

**Results:**

ASD group had significantly decreased callosal *f*_axon_ and *D*_axon_ (*p* = .01 and *p* = .045), particularly in the midbody, isthmus, and splenium. Regression analysis showed that variation in DKI metrics, primarily in the mid and posterior callosal regions explained up to 70.7% of the variance in processing speed scores for TD (*p* = .001) but not for ASD (*p* > .05).

**Conclusion:**

Decreased DKI metrics suggested that ASD may be associated with axonal deficits such as reduced axonal caliber and density in the corpus callosum, especially in the mid and posterior callosal areas. These data suggest that impaired interhemispheric connectivity may contribute to decreased processing speed in ASD participants.

**Electronic supplementary material:**

The online version of this article (10.1186/s13229-018-0245-1) contains supplementary material, which is available to authorized users.

## Background

Although the characterization of autism has evolved since Leo Kanner’s first identification of the syndrome in 1943, the specific genetic and neuronal components that contribute to the various symptoms are still largely unknown. In the 5th edition of Diagnostic and statistical manual of mental disorders [4], autism spectrum disorder (ASD) describes a range of neurodevelopmental disorders that are characterized by restrictive/repetitive behaviors and difficulties in social interaction and communication [18, 40].

The literature suggests that the pervasive deficits in social communication and cognitive performance observed in ASD reflect disruptions in neural connectivity throughout the brain, including both atypical functional connectivity [5, 8, 13, 33, 35] and alterations in the properties of white matter pathways [7, 33, 53, 62]. Numerous findings from histological and imaging studies have implicated the corpus callosum, the most prominent cerebral white matter tract, in the pathophysiology of ASD [6, 19, 25, 43, 46, 56]. Situated in the center of the mammalian brain, the corpus callosum consists of around 200 million fibers that radiate bilaterally to various cortical regions and facilitate inter-hemispheric communication [55]. Several schemes have been proposed to partition the corpus callosum into segments connecting distinct brain regions. A parcellation proposed by Witelson [61] segments callosum in five parts in the mid-sagittal plane. Hardan et al. [22] used the Witelson subdivisions but further divided the corpus callosum into seven segments, whereas Hofer and Frahm [26] proposed a revised five-division scheme based on tractography data and callosal connectivity to cortical regions. Specifically, anterior parts of the corpus callosum, including rostrum and genu, connect prefrontal and premotor areas; anterior and posterior midbody segments connect motor and somatosensory areas; the isthmus links superior temporal and posterior parietal regions; and the most posterior callosal segment, the splenium, connects superior parietal, occipital, and inferior temporal regions [14, 22, 26, 27, 50].

Prior morphometric and diffusion imaging studies have revealed that, compared to neuro-typical participants, autistic subjects have smaller volumes of both the entire callosum [2, 23] and callosal sub-regions [22, 56], specifically decreased white matter density [12, 53, 59], and increased diffusivity [7, 38, 47]. These findings support the aberrant neural connectivity hypothesis of ASD [5, 8, 34], which posits that the social and cognitive symptoms by which ASD is defined are related to a decrease in neural connectivity resulting from pervasive abnormalities in long-range white matter pathways. Abnormal myelin development in the corpus callosum has also been proposed [20] although evidence supporting this hypothesis remains incipient.

Despite general agreement on the involvement of the corpus callosum in ASD pathology, specific results diverge. This may be in part due to differences across studies in imaging and analysis methods. Nevertheless, the precise morphological variations as well as how changes in fiber tracts and microscopic features in the callosum are related to the diverse symptoms in ASD are still open for discussion [35, 36, 53]. The advent of more intricate diffusion imaging techniques and mathematical models may provide a more refined description of white matter microstructure and its relationship to symptoms and behavior.

Traditional diffusion tensor imaging (DTI) is based on a simplified Gaussian distribution of water diffusion that is problematic when encountering complex microscopic organizations in which diffusion may be non-Gaussian. Diffusional kurtosis imaging (DKI) aims to detect non-Gaussian diffusive behavior by introducing kurtosis as a marker reflective of tissue heterogeneity [51]. Moreover, the interpretation of DKI metrics can be further augmented by employing multi-compartment models for white matter that yield more detailed structural properties [17, 45]. For example, the two-compartment model separates MR signal contributions from intra-axonal and extra-axonal water while neglecting myelin water contributions, as they are not detectable with the imaging parameters employed in typical diffusion imaging studies [16, 17].

In two-compartment DKI, diffusion in each compartment is measured by a different diffusion tensor. One of the principal parameters of interest is the axonal water fraction (*f*_axon_), which is the fraction of MRI-visible intra-axonal water relative to total intra- and extra-axonal water. The model also provides diffusivity metrics that characterize the properties of the two compartments: intra-axonal diffusivity, *D*_axon_, which is assumed to measure along the axonal axis, and extra-axonal axial (AD_extra_) and radial (RD_extra_) diffusivities, which are assumed to reflect water diffusion in the extra-cellular space along and perpendicular to axons, respectively [38]. Each of these parameters gives more detailed information about specific properties of white matter axonal packing than DTI. *f*_axon_ changes with the water signal inside axons so that it is related to density of axonal packing and axonal caliber. The more densely packed or larger axons are, the higher *f*_axon_ is. *D*_axon_ reflects intra-axonal microscopic properties including variations in the size and number of intra-axonal structures such as microfilaments or mitochondria. In the extra-axonal environment, AD_extra_ is assumed to reflect structures such as oligodendrocytes and astrocytes or extracellular inflammation. RD_extra_ is influenced by myelination since the myelin sheath impedes the diffusion of water perpendicular to the axon packing direction [9, 17].

In the current study, we employed DKI along with the two-compartment model to compare intra- and extra-axonal diffusion properties in five discrete callosal areas in typically developing controls versus age- and IQ-matched ASD subjects. Our goal was to increase specificity and sensitivity in identifying callosal microstructural deficits in ASD. We also investigated the relationship between callosal white matter properties and cognitive measurements indexing information processing based on two observations: (1) the corpus callosum is known to support processing speed [10, 44], and (2) impaired processing speed has been consistently reported in ASD [21, 54]. Thus, we tested whether indices of processing speed can be predicted by callosal white matter DKI properties.

## Methods

### Participants and design

Seventeen typically developing (TD) controls and 16 individuals aged 18 to 25 years old with a diagnosis of ASD and no co-occurring intellectual disability (ID) (i.e., IQ > 80) participated in the study. The study was approved by the NYU School of Medicine Institutional Review Board. All participants provided informed consent at the time of their visit. TD participants had reported no personal or family history of ASD or other psychiatric conditions. None of the participants reported previous head injury or organic brain damage.

Autism Diagnostic Observation Schedule (ADOS) [41] and Autism Diagnostic Interview-Revised (ADI-R) [42] were used to confirm diagnosis in ASD participants. Four of the ASD participants had either co-occurring or history of psychiatric problems according to self-report, including anxiety, depression, post-traumatic stress disorder, obsessive-compulsive disorder, and history of attention-deficit/hyperactivity disorder.

All participants received IQ assessments based on Wechsler Adult Intelligence Scale-III (WAIS-III) [58]. The WAIS-III IQ scores were used to assess cognitive ability and confirm the lack of ID in all participants. The WAIS-III generates a Full Scale IQ (FSIQ), which further includes four indices: Verbal Comprehension Index (VCI), Perceptual Organization Index (POI), Working Memory Index (WMI), and Processing Speed Index (PSI). Decreased processing speed is one of the more consistent findings in ASD [21, 54], and it has been proposed to be related to abnormalities in white matter pathways [38]. The two subtests composing the WAIS-III PSI, Digit Symbol-Coding (DigitSC) and Symbol Search (SS), were further examined to assess if they are governed by different relationships to callosal microstructural properties.

Anatomical T1-weighted (T1w) images and diffusion data were collected for all participants. Several diffusion metrics were calculated as previously described [38] and employed to compare white matter properties in ASD participants versus TD individuals. Multivariate regression analyses of processing speed-related indices with the diffusion metrics were conducted to explore the impact of white matter characteristics in the corpus callosum on cognitive performance.

### Data acquisition and processing

Diffusion imaging scans were performed on a 3T Siemens Trio System (Siemens, Erlangen, Germany) using a 12-channel array coil. Diffusion data was obtained using a twice-refocused diffusion-weighted echo planar imaging sequence with a GRAPPA parallel imaging factor of 2 and 24 reference lines. Between 55 and 60 slices were acquired using 2.3 × 2.3 × 2.3 mm^3^ isotropic voxels. Other imaging parameters included TE = 97 ms and TR = 8100 ms. Diffusion-weighted data was obtained for two diffusion weighting (*b* = 1000 and 2000 s/mm^2^) with 12 non-collinear encoding directions acquired for *b* = 1000 s/mm^2^ and 42 non-collinear encoding directions acquired for *b* = 2000 s/mm^2^. Each diffusion-weighted acquisition was repeated twice to increase signal-to-noise ratio. Ten images with *b* = 0 s/mm^2^ were also collected. To describe B0-field inhomogeneities, field map images consisting of one phase image and one corresponding magnitude image were acquired coplanar to the diffusion images using a Siemens provided sequence. The product sequence acquired two gradient echo images with different TE (TE1 = 8 ms and TE2 = 10.46 ms) to produce phase and magnitude images that map B0-field inhomogeneities.

The images were preprocessed and corrected for motion and distortions from eddy currents and magnetic field inhomogeneities using in-house developed code in Matlab (Mathworks, Natick, Massachusetts), Interactive Data Language (IDL, Exelis Visual Information Solutions, Boulder, Colorado), and FMRIB Software Library (FSL4.1, http://www.fmrib.ox.ac.uk/fsl). The diffusion data was first smoothed using a Gaussian filter with *σ* = 1.2 mm to improve the quality of DKI fitting. Eddy currents and motion were corrected with in-house written scripts using FSL flirt [31, 32]. FSL prelude, fugue, and fslmaths were used to apply the field map correction to the diffusion images [48]. Several steps were employed as recommended by the FSL guide, i.e., transforming the phase image to radians and unwrapping it, calculating and regularizing the shiftmap, and aligning the shiftmap to the diffusion images and unwarping it. The quality of the correction was visually assessed by overlapping the FA image onto the T1-weighted image that was registered to the diffusion space. A good correspondence between these two images was obtained in all subjects.

Apart from distortion correction, all images were visually examined for signal dropouts resulting from cerebrospinal fluid pulsations or blurring from movement. Both slices and volumes affected by signal dropout or blurring from either motion or cerebrospinal fluid pulsations were removed from the analyses. In all but two participants, the presence of such artifacts was minimal with at most one or two volumes removed from the total of 118 collected. Thus, we estimated that removal of this data had a negligible effect on the DKI fit. We note that in all of these cases, all encoding directions were represented since data was available for at least one of the two averages collected. Two data sets from the ASD group had more motion, and thus, approximately 12% data was removed. Again, motion occurred at different times during the two acquisitions, and thus, most encoding directions were available for analyses with only one or at most two encoding directions out of 54 lost. We note that the number of encoding directions employed here was well in excess of the minimum number of encodings needed for DKI (*n* = 21). Exclusion of the two more affected data sets from analyses did not affect between-group differences [38], and thus, data sets for all participants were included. Following visual image inspection, the two repeated acquisitions were combined using a weighted average approach that included only viable data.

The calculation of DKI metrics was conducted using in-house written software, which was based on the non-Gaussian water diffusion assumption that allows estimations of both diffusion and kurtosis tensors using a constrained linear least squares regression approach [52]. The kurtosis tensor data were used in the calculation of intra- and extra-axonal diffusivities, *D*_axon_, AD_extra_, and RD_extra_ and the axonal water fraction, *f*_axon_ (for details see [38]). The diffusion tensor obtained as part of the DKI fitting was used to derive FA and axial (AD) and radial diffusivity (RD) maps.

**Fig. 1:**
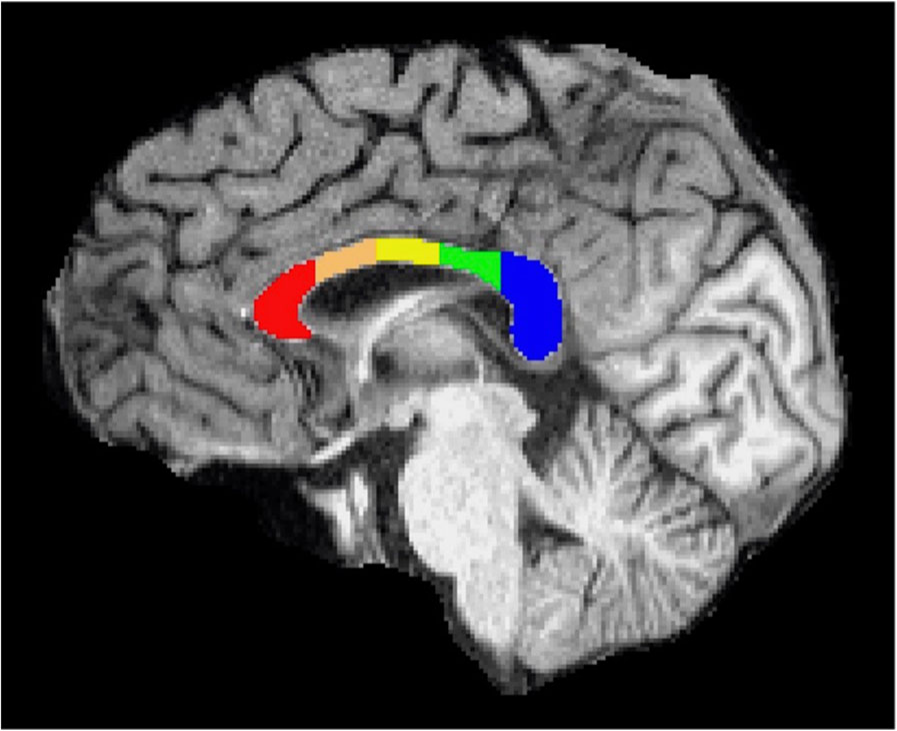
The five callosal regions examined in this work, depicted in mid-sagittal cross-section: segment 1—red, segment 2—orange, segment 3—yellow, segment 4—green, segment 5—blue

The T1w images along with FreeSurfer software (Desikan-Killiany atlas) were used to obtain a five-region of interest (ROI) segmentation of the midline corpus callosum in each subject (Fig. 1). These ROIs were registered to the diffusion data and employed to generate five callosal segments that extended from the midline bilaterally to the corona radiata. To limit the effects of partial volume averaging with neighboring cerebrospinal fluid, we limited each ROI to voxels with mean diffusivity (MD) smaller than 1.5 × 10^−3^ mm^2^/s, based on previous work describing the normal range of MD values in the human brain (e.g., [11]). Mean values of each of the diffusion metrics were derived for each of the five resulting callosal segments.

### Statistical analyses

Statistical analyses and graphing were conducted with SPSS 20.0 (IBM, Armonk, NY) and Matlab. One SS value was missing, which resulted in a missing value in the corresponding PSI score. We replaced the SS value with a value imputed by regression from DigitSC scores (i.e., the other subtest in PSI). Between-group differences for demographic characteristics and IQ scores were analyzed individually using *t* tests. Two-way analysis of variance (ANOVA) was applied to the diffusion metrics with the five callosal segments included as a blocking factor to account for the lack of statistical independence among DKI measures derived for the five segments per participant. Equal variance was assumed for DKI measures, and Levene’s test was used to support the assumption. Separate two-way ANOVAs were performed for each of the four DKI metrics, and overall between-group differences across segments were assessed. Post hoc pairwise comparisons were then used to further evaluate differences in callosal segments. Correction for multiple comparisons was conducted using the Benjamini-Hochberg approach. Given the relatively small sample size employed in this study, a moderate approach that accounted for the five different regions examined was employed to minimize type I errors while maintaining statistical power. Uncorrected *p* values are presented in the results, with values reaching significance after correction highlighted with an asterisk.

Building on findings from ANOVA, multivariable regression analyses for processing speed scores and DKI metrics in the callosal segments were run to test if processing speed can be predicted by diffusion metrics in each segment of the corpus callosum with DigitSC and SS scores used as outcome variables, and *f*_axon_, *D*_axon_, AD_extra_, and RD_extra_ derived from five callosal segments used as predictors. For comparison with other datasets, bivariate correlations are reported in Additional file 1.

In all model fitting, we controlled for the number of callosal voxels by including it at the first step as a covariate. Previous work [57] has shown that the size of corpus callosum influences the degree of partial volume averaging with nearby structures (e.g., cerebrospinal fluid) and thus may induce artificial alterations of intrinsic callosal diffusion properties. Therefore, to counteract the effect of partial volume, we rejected voxels with abnormally high MD (as described in the “Data acquisition and processing” section) and added the number of voxels as a covariate in our main analyses. We note that analyses conducted without controlling for the number of voxels yielded similar results and are thus not included here. Subject age was also not included as a covariate since no significant relationship was found in preliminary analyses between age and DKI measures, and WAIS-III scores account for age in the scoring process [58]. As preliminary data analysis revealed different patterns of correlation in the two groups, regression analyses were carried out separately within each group.

## Results

### Demographic data and IQ score

There were no significant between-group differences in age (*p* = .678), handedness (*p* = .809), or Full score IQ (*p* = .143). However, in one of the four indices in the intelligence test, PSI, ASD participants (93.44 ± 18.37) scored substantially lower than TD controls (108.71 ± 14.28) (*p* = .012), with significant group differences found in both subtest scores—DigitSC (*p* = .028) and SS (*p* = .008) (Table 1).

**Table 1:** Summary of demographic and IQ data for the TD and ASD groups. Significant group differences were found in processing speed and its two component subtests, DigitSC and SS

	TD (*n* = 17)	ASD (*n* = 16)	*p* value
Age	21.71 ± 2.14	21.38 ± 2.39	.678
Handedness	14.53 ± 3.59	14.81 ± 3.04	.809
Full score IQ	116.65 ± 11.98	108.88 ± 17.39	.143
Verbal comprehension index	119.18 ± 13.14	115.13 ± 23.75	.545
Perceptual organization index	113.76 ± 15.34	107.56 ± 12.06	.208
Working memory index	107.76 ± 12.39	104.56 ± 15.40	.514
Processing speed index	108.71 ± 14.28	93. 44 ± 18.37	.012*
DigitSC	11.12 ± 2.71	8.13 ± 4.59	.028*
SS	12.41 ± 2.85	9.56 ± 2.92	.008*

*p < .05

### Diffusion metrics

**Fig. 2:**
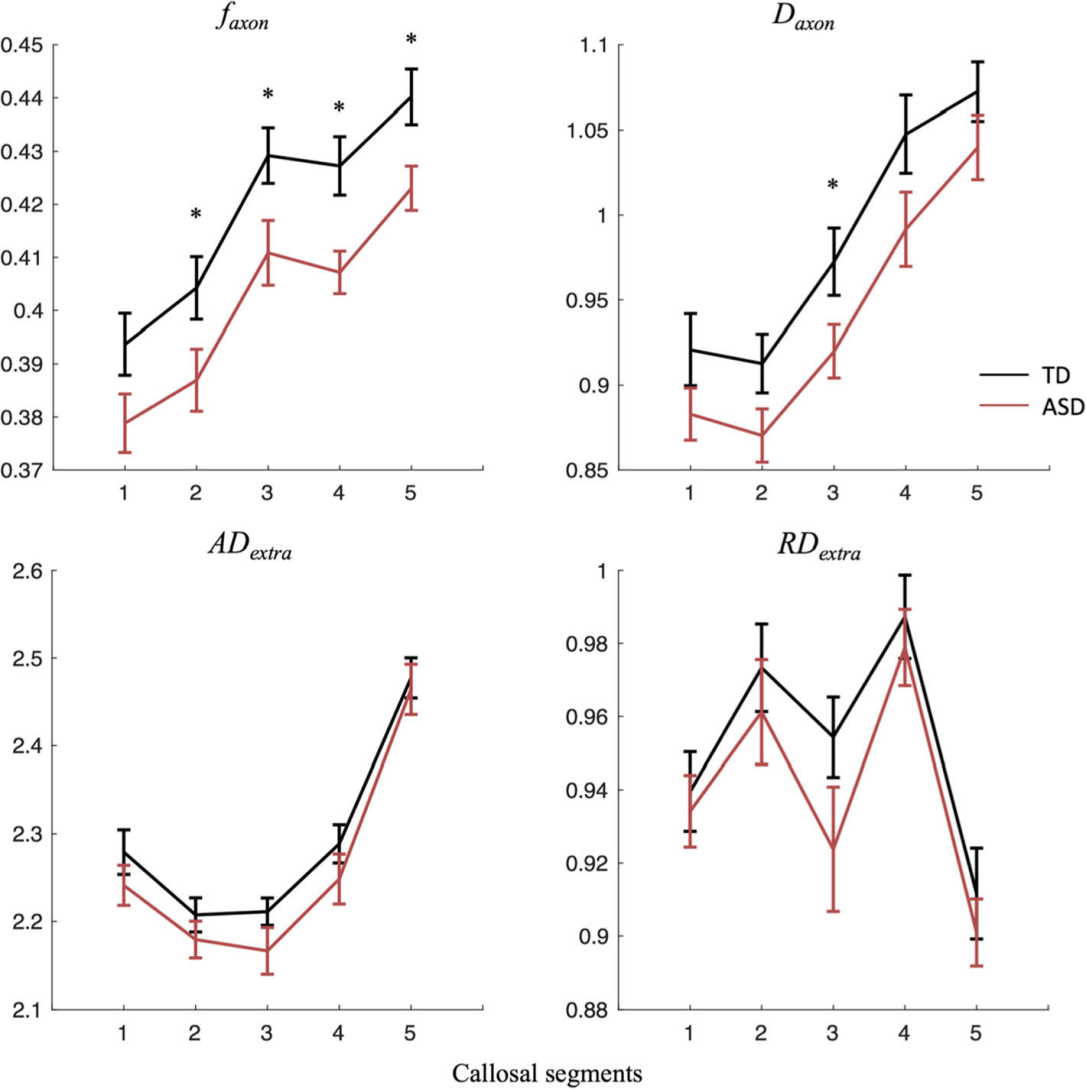
Group comparisons for DKI metrics by segment, with segment 1 to 5 representing anterior to posterior callosal region (*p < .05). Black and red lines represent TD controls and ASD participants, respectively. Error bars depict standard error

Two-way ANOVA revealed significant group differences for both *f*_axon_ (*p* = .010) and *D*_axon_ (*p* = .045). As demonstrated in Fig. 2, the TD group had *f*_axon_ and *D*_axon_ values higher than the ASD group, and the effect seemed to encompass all segments of the corpus callosum. Main effect of segment was also significant for all four diffusion metrics (*p* < .0001), suggesting varying diffusion properties across the five callosal segments. Post hoc analyses revealed differences between groups for *f*_axon_ in callosal segments 2 to 5 and for *D*_axon_ in callosal segment 3. After correcting for multiple comparisons, group differences in segment 3 to 5 for *f*_axon_ were found to be significant (Table 2). AD_extra_, RD_extra_, and the number of callosal voxels did not differ across groups. No significant between-group differences were noted in any of the traditional diffusion tensor metrics (Additional file 1: Figure S1).

**Table 2:** Summary of group differences from two-way ANOVA for DKI measures, including number of volumes, *f*_axon_, *D*_axon_, AD_extra_, and RD_extra_ across callosal body and by callosal segment (*F*, uncorrected *p*, and effect size partial *η*^*2*^ values are reported)

	TD	ASD	*F*(1, 31)	*p*	*η* ^*2*^
*f*_axon_	.422 ± .020	.405 ± .014	7.47	.010*	.19
*f*_axon_ in segment 1	.394 ± .024	.379 ± .022	3.42	.074	.10
*f*_axon_ in segment 2	.404 ± .024	.387 ± .023	4.34	*.046*	.12
*f*_axon_ in segment 3	.429 ± .022	.411 ± .025	4.30	.029*	.15
*f*_axon_ in segment 4	.427 ± .023	.407 ± .016	8.52	.006*	.22
*f*_axon_ in segment 5	.440 ± .021	.423 ± .017	6.51	.016*	.17
*D*_axon_	1.001 ± .067	.961 ± .052	4.26	.045*	.12
*D*_axon_ in segment 1	.921 ± .087	.883 ± .061	2.06	.161	.06
*D*_axon_ in segment 2	.913 ± .071	.870 ± .062	3.27	.080	.10
*D*_axon_ in segment 3	.972 ± .082	.920 ± .063	4.00	*.048*	.12
*D*_axon_ in segment 4	1.047 ± .094	.992 ± .088	3.10	.088	.09
*D*_axon_ in segment 5	1.073 ± .072	1.040 ± .076	1.62	.213	.05
AD_extra_	2.338 ± .068	2.313 ± .083	1.76	.194	.05
RD_extra_	.940 ± .041	.926 ± .032	0.94	.339	.03
Number of volumes	2854 ± 718	2882 ± 542	0.02	.901	.00

Italics indicate *p* < .05 uncorrected

*Significant result after correcting for multiple comparisons using Benjamini-Hochberg procedure with false discovery rate (FDR) set at 5%

**Fig. 3:**
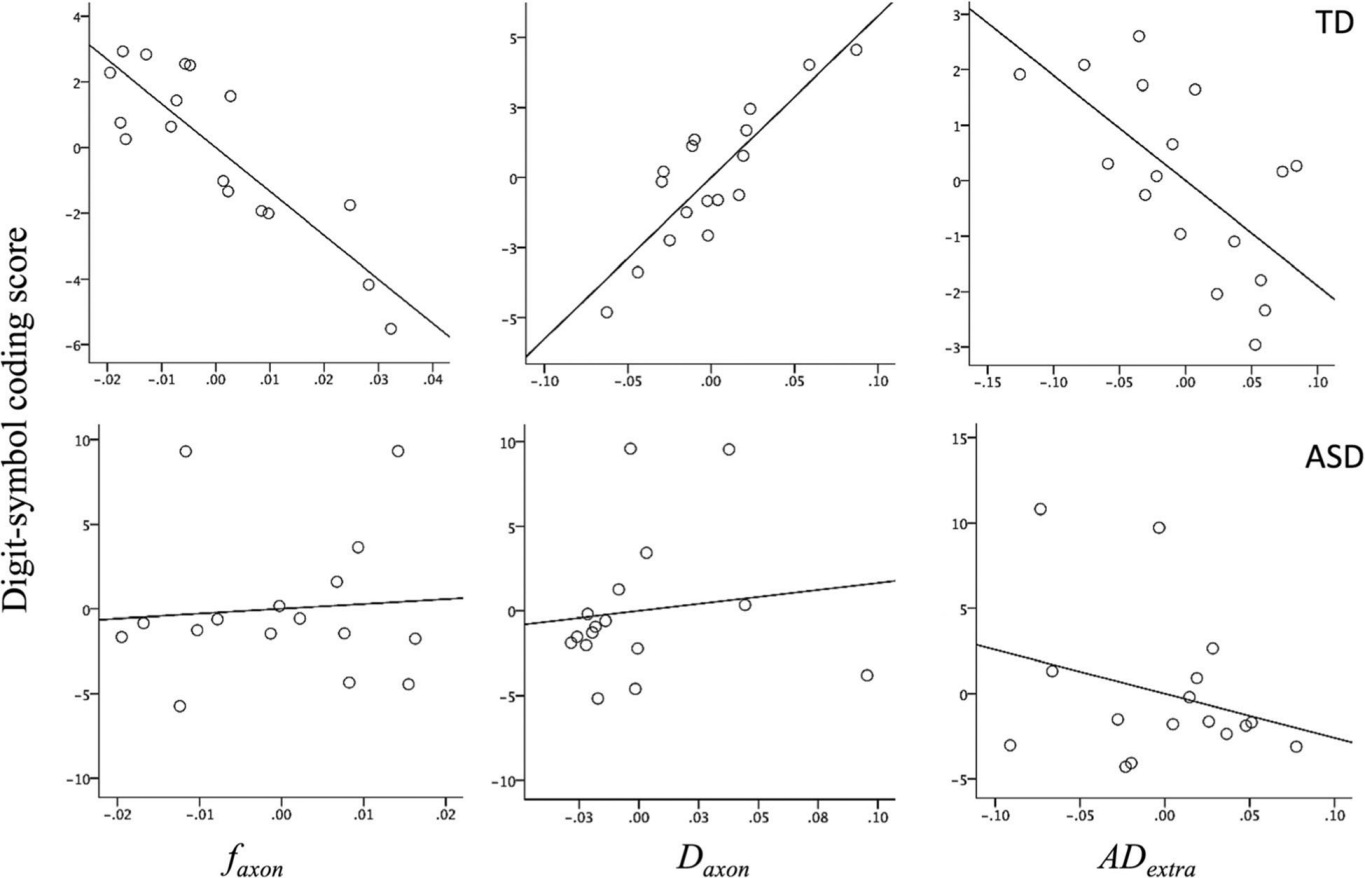
Partial correlation plots for one of the regression models describing digit-symbol coding score dependence on callosal diffusion metrics. Top: model 5 for TD group (Table 3), bottom: equivalent model for the ASD group (Tables 4). Significant partial correlations are noted in the TD but not in the ASD group

Regression analyses showed that DigitSC can be significantly predicted by microstructural callosal properties in TD but not in the ASD group (Tables 3 and 4; Fig. 3). More specifically, regression models with DKI metrics from segments 3, 4, and 5 were found to be significant in characterizing changes in DigitSC scores in TD (Table 3). In addition, a strong relationship was found between SS scores and *f*_axon_ and *D*_axon_ as well as the number of callosal voxels in segment 2 for TD. No similar relationships were found in ASD subjects (Table 4).

**Table 3:** Regression models for TD group describing processing speed scores’ dependence on *f*_axon_ and *D*_axon_ in the mid and posterior segments, controlling for the number of voxels (NumVox) in that segment. Adjusted *R*^2^ (i.e., *R*^2^ adjusted for the number of predictors in the model) and uncorrected *p* values are reported

Model	Predictors		Sig. (predictors)	Sig. (model)	*R*^2^ (model)
1 (DigitSC)	Covariate	NumVox (seg1)	.255	.215	.179
	Investigated predictors	*f*_axon_ (seg1)	.084		
		*D*_axon_ (seg1)	.120		
		AD_extra_ (seg1)	.416		
		RD_extra_ (seg1)	.243		
2 (DigitSC)	Covariate	NumVox (seg2)	.511	.312	.100
	Investigated predictors	*f*_axon_ (seg2)	.284		
		*D*_axon_ (seg2)	.080		
		AD_extra_ (seg2)	.213		
		RD_extra_ (seg2)	.429		
3 (DigitSC)	Covariate	NumVox (seg3)	.663	.025*	.385
	Investigated predictors	*f*_axon_ (seg3)	.014*		
		*D*_axon_ (seg3)	.053		
4 (DigitSC)	Covariate	NumVox (seg3)	.986	.035*	.382
	Investigated predictor	RD_extra_ (seg3)	.019*		
5 (DigitSC)	Covariate	NumVox (seg4)	.088	.016*	.429
	Investigated predictors	*D*_axon_ (seg4)	.003*		
		AD_extra_ (seg4)	.056		
6 (DigitSC)	Covariate	NumVox (seg5)	.005*	.001*	.707
	Investigated predictors	*f*_axon_ (seg5)	.001*		
		*D*_axon_ (seg5)	.002*		
		AD_extra_ (seg5)	.012*		
7 (SS)	Covariate	NumVox (seg1)	.339	.399	.040
	Investigated predictors	*f*_axon_ (seg1)	.461		
		*D*_axon_ (seg1)	.748		
		AD_extra_ (seg1)	.719		
		RD_extra_ (seg1)	.650		
8 (SS)	Covariate	NumVox (seg2)	.004*	.020*	.407
	Investigated predictors	*f*_axon_ (seg2)	.026*		
		*D*_axon_ (seg2)	.095		
9 (SS)	Covariate	NumVox (seg3)	.269	.182	.212
	Investigated predictors	*f*_axon_ (seg3)	.752		
		*D*_axon_ (seg3)	.180		
		AD_extra_ (seg3)	.289		
		RD_extra_ (seg3)	.308		
10 (SS)	Covariate	NumVox (seg4)	.253	.366	.044
	Investigated predictors	*f*_axon_ (seg4)	.378		
		*D*_axon_ (seg4)	.354		
		AD_extra_ (seg4)	.928		
		RD_extra_ (seg4)	.921		
11 (SS)	Covariate	NumVox (seg1)	.190	.197	.049
	Investigated predictors	*f*_axon_ (seg1)	.556		
		*D*_axon_ (seg1)	.564		
		AD_extra_ (seg1)	.985		
		RD_extra_ (seg1)	.939		

*p < .05

**Table 4:** Regression models for ASD group describing processing speed scores’ lack of dependence on *f*_axon_ and *D*_axon_ in the mid and posterior segments. As in the analyses reported in Table 3, analyses controlled for the number of voxels (NumVox) in the designated segment. Adjusted *R*^2^ (i.e., *R*^2^ adjusted for the number of predictors in the model) and uncorrected *p* values are reported

Model	Predictors		Sig. (predictors)	Sig. (model)	*R*^2^ (model)
1 (DigitSC)	Covariate	NumVox (seg1)	.997	.254	.160
	Investigated predictors	*f*_axon_ (seg1)	.785		
		*D*_axon_ (seg1)	.110		
		AD_extra_ (seg1)	.138		
		RD_extra_ (seg1)	.587		
2 (DigitSC)	Covariate	NumVox (seg2)	.614	.339	.023
	Investigated predictors	*f*_axon_ (seg2)	.371		
		*D*_axon_ (seg2)	.950		
		AD_extra_ (seg2)	.498		
		RD_extra_ (seg2)	.836		
3 (DigitSC)	Covariate	NumVox (seg3)	.549	.738	− .178^1^
	Investigated predictors	*f*_axon_ (seg3)	.344		
		*D*_axon_ (seg3)	.894		
		AD_extra_ (seg3)	.220		
		RD_extra_ (seg3)	.630		
4 (DigitSC)	Covariate	NumVox (seg4)	.407	.221	.190
	Investigated predictors	*f*_axon_ (seg4)	.061		
		*D*_axon_ (seg4)	.812		
		AD_extra_ (seg4)	.099		
		RD_extra_ (seg4)	.524		
5 (DigitSC)	Covariate	NumVox (seg1)	.970	.870	− .275^1^
	Investigated predictors	*f*_axon_ (seg1)	.748		
		*D*_axon_ (seg1)	.830		
		AD_extra_ (seg1)	.509		
		RD_extra_ (seg1)	.723		
6 (SS)	Covariate	NumVox (seg1)	.670	.372	.046
	Investigated predictors	*f*_axon_ (seg1)	.906		
		*D*_axon_ (seg1)	.261		
		AD_extra_ (seg1)	.143		
		RD_extra_ (seg1)	.963		
7 (SS)	Covariate	NumVox (seg2)	.675	.362	.033
	Investigated predictors	*f*_axon_ (seg2)	.160		
		*D*_axon_ (seg2)	.550		
		AD_extra_ (seg2)	.813		
		RD_extra_ (seg2)	.386		
8 (SS)	Covariate	NumVox (seg3)	.269	.339	.066
	Investigated predictors	*f*_axon_ (seg3)	.752		
		*D*_axon_ (seg3)	.180		
		AD_extra_ (seg3)	.289		
		RD_extra_ (seg3)	.308		
9 (SS)	Covariate	NumVox (seg4)	.253	.375	.008
	Investigated predictors	*f*_axon_ (seg4)	.378		
		*D*_axon_ (seg4)	.354		
		AD_extra_ (seg4)	.928		
		RD_extra_ (seg4)	.921		
10 (SS)	Covariate	NumVox (seg1)	.190	.394	.052
	Investigated predictors	*f*_axon_ (seg1)	.556		
		*D*_axon_ (seg1)	.564		
		AD_extra_ (seg1)	.985		
		RD_extra_ (seg1)	.939		

^1^Negative adjusted *R*^2^ values indicate poor fit of the data

Since a large variation in IQ was noted within the ASD group, with two participants having extremely high IQ, we tested if presence of outliers influenced the results by repeating analyses without them. Overall results remained unchanged (Additional file 1: Figures S2 and S3).

## Discussion

### Callosal abnormalities in ASD

Compared with typically developing controls, ASD subjects scored significantly lower in the WAIS-III processing speed index than TD, as previously reported [21, 54]. This effect was reliably observed with both subtests, Digit Symbol Coding (DigitSC) and Symbol Search (SS). Correspondingly, DKI metrics *f*_axon_ and *D*_axon_ were also significantly decreased in ASD compared with TD. This effect was primarily driven by differences in specific callosal segments: for *f*_axon_, significant between-group differences were found in the midbody and the posterior regions of the corpus callosum, and for *D*_*axon*_, in the midbody.

These results suggest differences in axonal caliber and/or axonal packing of callosal regions in ASD subjects. Since no significant group-differences were found for the two extra-axonal DKI measures in the current study, the microarchitecture of the extracellular environment, including the organization of glial cells and myelination, may not be severely affected in the current ASD sample [38], although further work including more direct metrics of myelin content are needed to address this hypothesis. Moreover, future studies should assess between-group differences across a range of ages to understand the potential impact of development on microstructural white matter features (e.g., axonal density, myelin content) and their relative contribution to ASD deficits.

Incorporating existing findings of axonal organization and topographic connectivity of the corpus callosum, current results suggest that ASD may be characterized by reduced axonal caliber in the corpus callosum and differences in the axonal milieu, with larger axons in the middle and posterior callosal regions being the most affected [1]. Smaller diameter fibers in the corpus callosum may interfere with interhemispheric connections of the motor, occipital, and temporal cortices [26], thus disrupting their functioning and altering connectivity patterns in the whole brain.

### Potential network dysfunctions in ASD

Multiple regression analyses showed that while DKI metrics could explain an impressive proportion of the variance in processing speed for TD subjects, the same did not hold for the ASD group. This divergence may indicate alterations in the underlying mechanisms supporting processing speed in ASD individuals.

In the TD group, an overall negative correlation was found between *f*_axon_ and DigitSC (Fig. 3 and Additional file 1: Figure S2), which is in line with previous findings showing negative correlations between DTI-based fractional anisotropy metric (FA) and performance IQ score during adolescence and young adulthood [3, 28]. Hutchinson et al. [28] hypothesized that processing of easy tasks often relies on intrahemispheric connections for the benefit of fast information transfer, while complex tasks need to recruit interhemispheric interaction to balance processing accuracy and efficiency. In short, the corpus callosum appears to be responsible for the effective distribution of processing load when it exceeds intrahemispherical capacity either due to increased load or impaired intrahemispheric communication. In TD controls, the lower *f*_axon_ in the corpus callosum may reflect more proficient intrahemispheric connections, which is associated with higher performance IQ [3, 28]. Furthermore, processing speed relationships with two other DKI metrics (*D*_axon_ and RD_extra_) were also noted. Together, these data suggest that multiple white matter features may contribute to processing speed and thus may need to be accounted for when predicting cognitive ability.

In the ASD group, the decreased *f*_axon_ and *D*_axon_ and the lack of an association between processing speed and callosal diffusion metrics have several implications. First, it suggests that processing speed within the ASD group, as tested with current indices, might be primarily contributed by intrahemispheric associations instead of interhemispheric callosal connections. This hypothesis is indirectly supported by findings by Lazar et al. [38], who found positive correlation between DigitSC score and several intra-hemispheric association tracts in ASD subjects. However, the intrahemispheric pathways in ASD were also found to have decreased *f*_axon_ and *D*_axon_ [38]. Thus, altered callosal microstructure in ASD may compromise the ability of the corpus callosum to distribute the processing load across hemispheres and compensate for likely lower intrahemispheric capacity, contributing to lower scores in processing speed indices. Second, the ASD group may be more heterogeneous, which combined with our relative small sample size may lead to weaker associations between imaging and cognitive metrics. There is increasing evidence that ASD may involve different levels of disrupted excitatory/inhibitory circuits [24, 63] and noisy, unreliable neural signals [60]. These perspectives suggest it may be fruitful to identify ASD subgroups to better understand ASD pathophysiology, and such work will require substantially larger samples.

In the current study, we found altered white matter microstructure in the mid and posterior regions of the corpus callosum but not in the anterior callosum, which is consistent with hypotheses that differences in brain organization in ASD stem from deficits in brain regions responsible for lower-order processing (e.g., sensation perception and motor execution). As children mature, abnormalities in lower-order processing networks would affect regions responsible for higher-order cognition [39]. In the current sample of ASD, inter-hemispheric deficits were detected in lower-order interhemispheric networks (e.g., motor, visual, auditory). Compared to low-functioning ASD, ASD with no ID may be characterized by a developmental course of less pervasive lower deficit spread across networks, resulting in more efficient higher-order networks, the mechanisms of which may be worth further investigation.

### Limitations and future directions

The sample size of the current study was relatively small, which decreased statistical power. This prompted us to choose a less strict correction procedure for multiple comparison problem to minimize false discovery rate while preserving statistical power. Thus, although the observed effects were robust, replication in larger samples is needed. Furthermore, as is generally the case in autism brain imaging studies, we only included young adult ASD male subjects without ID and age-matched male controls [18]. Therefore, extension of this work to females, children, and a broader range of functioning will be important future goals.

We also note that the white matter model we used is based on idealized assumptions (e.g., axons are parallel within a voxel) [17] and fitting of the diffusion data to multi-compartment models is not trivial [29]. Despite these potential shortcomings, initial studies comparing imaging and histology data suggest that the results of these models are consistent with underlying microstructure. Both Jelescu et al. [30] and Falangola et al. [15] compared imaging and histology results in cuprizone mouse models and reported that correlations between metrics reflective of axonal density (*f*_axon_) and myelination (RD_extra_) and their histological counterparts follow the expected pattern. In these studies, *f*_axon_ was significantly associated with the electron microscopy-derived axonal water fraction, but not with myelin-related histological metrics (myelin volume fraction and g-ratio). By contrast, as expected, RD_extra_ followed the opposite pattern, correlating with myelin-related histological metrics but not with the histology-derived axonal water fraction. In addition, findings here are consistent with a previous study employing the same sample [38], which focused on a different set of white matter tracts and used a voxel-based approach, tract-based spatial statistics [49], in analyzing the data. As imaging techniques and the accuracy of scientific models improve [37], we can be optimistic that our understanding of the mechanisms for complex psychiatric disorders will be expanded and refined.

## Conclusion

Diffusional kurtosis imaging and a two-compartment model suggest reduced axonal caliber of large-diameter axons in the mid and posterior regions of the corpus callosum in young male adults with ASD without ID, which may result in atypical integration of neural signals across brain regions manifesting in impaired processing speed. We infer that weaker callosal interhemispheric connections in young adults with ASD lead to greater reliance of processing speed on intrahemispheric associations. Considering the heterogeneity of behavioral patterns and developmental trajectories in ASD, this hypothesis will need to be confirmed through longitudinal studies with larger sample sizes.

## Additional file

Additional file 1: **Figure S1.** Traditional diffusion metrics for TD and ASD group (fractional anisotropy, FA; axial diffusivity, AD; radial diffusivity, RD). No significant group difference was found in FA, AD, or RD. The DTI results are comparable to previously published studies (Travers et al., 2012) [53]. Our data suggested increased sensitivity of the DKI metrics to group differences compared to DTI ones. **Figure S2.** Scatter plots showing bivariate relationships between DKI metrics and DigitSC score for TD and ASD group. Each row represents one DKI metrics while each column shows results for different segments. Plots where correlations between diffusion metrics and DigitSC reached *p* values smaller than 0.05 for the TD group are marked with a thicker black border. We note that bivariate correlations were relatively weaker compared to multivariable regression models although they did follow a similar trend. No significant correlations are noted in the ASD group. **Figure S3.** Scatter plots showing bivariate relationships between DKI metrics and DigitSC score for TD and ASD group, without the two ASD outliers with high DigitSC scores. Each row represents one DKI metrics while each column shows results for different segments. Plots where correlations between diffusion metrics and DigitSC reached *p* values smaller than 0.05 for the TD group are marked with a thicker black border. No significant correlations are noted in the ASD group. (ZIP 1620 kb)

## Abbreviations

AD_extra_: Extra-axonal axial diffusivity
ANOVA: Analysis of variance
ASD: Autism spectrum disorders
*D*_axon_: Intra-axonal diffusivity
DigitSC: Digit symbol coding index
DKI: Diffusional kurtosis imaging
DTI: Diffusion tensor imaging
*f*_axon_: Fraction of intra-axonal water
ID: Intellectual disability
MD: Mean diffusivity
PSI: Processing speed index
RD_extra_: Extra-axonal radial diffusivity
ROI: Region of interest
SS: Symbol search index
TD: Typically developing controls
