# Supplementary material for: Diffusional kurtosis imaging of the corpus callosum in autism
Source: Mol Autism. 2018 Dec 13;9:62. doi: 10.1186/s13229-018-0245-1 (PMC6293510; doi:10.1186/s13229-018-0245-1)
Supplement: Supplementary file 1 — Figure S1. Traditional diffusion metrics for TD and ASD group (fractional anisotropy, FA; axial diffusivity, AD; radial diffusivity, RD). No significant group difference was found in FA, AD, or RD. The DTI results are comparable to previously published studies (Travers et al., 2012) [53]. Our data suggested increased sensitivity of the DKI metrics to group differences compared to DTI ones. Figure S2. Scatter plots showing bivariate relationships between DKI metrics and DigitSC score for TD and ASD group. Each row represents one DKI metrics while each column shows results for different segments. Plots where correlations between diffusion metrics and DigitSC reached p values smaller than 0.05 for the TD group are marked with a thicker black border. We note that bivariate correlations were relatively weaker compared to multivariable regression models although they did follow a similar trend. No significant correlations are noted in the ASD group. Figure S3. Scatter plots showing bivariate relationships between DKI metrics and DigitSC score for TD and ASD group, without the two ASD outliers with high DigitSC scores. Each row represents one DKI metrics while each column shows results for different segments. Plots where correlations between diffusion metrics and DigitSC reached p values smaller than 0.05 for the TD group are marked with a thicker black border. No significant correlations are noted in the ASD group. (ZIP 1620 kb) [file 13229_2018_245_MOESM1_ESM.zip › Fig_S3.docx]

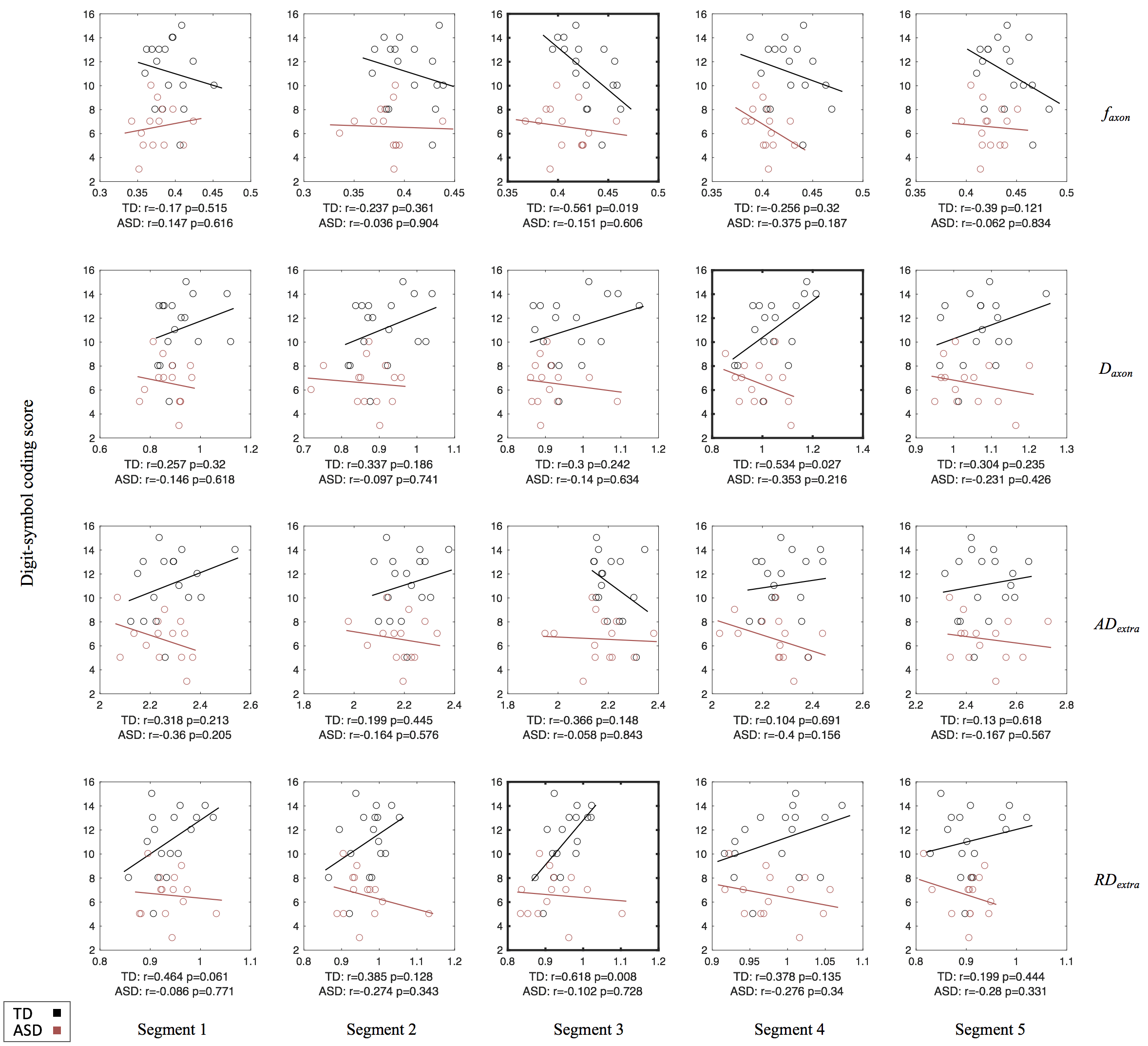


*Figure S3.* Scatter plots showing bivariate relationships between DKI metrics and DigitSC score for TD and ASD group, without the two ASD outliers with high DigitSC scores. Each row represents one DKI metrics while each column shows results for different segments. Plots where correlations between diffusion metrics and DigitSC reached *p*-values smaller than 0.05 for the TD group are marked with a thicker black border. No significant correlations are noted in the ASD group.
